# Contraceptive use among women through their later reproductive years: Findings from an Australian prospective cohort study

**DOI:** 10.1371/journal.pone.0255913

**Published:** 2021-08-11

**Authors:** Melissa L. Harris, Nicholas Egan, Peta M. Forder, Jacqueline Coombe, Deborah Loxton

**Affiliations:** 1 Centre for Women’s Health Research, University of Newcastle, Newcastle, New South Wales, Australia; 2 Hunter Medical Research Institute, Newcastle, New South Wales, Australia; 3 Melbourne School of Population and Global Health, University of Melbourne, Parkville, Victoria, Australia; Osakidetza Basque Health Service, SPAIN

## Abstract

**Objective:**

Examine patterns of contraceptive use and contraceptive transitions over time among an Australian cohort of women through their later reproductive years.

**Study design:**

Latent Transition Analysis was performed using data on 8,197 women from the Australian Longitudinal Study on Women’s Health’s 1973–78 cohort to identify distinct patterns of contraceptive use across 2006, 2012 and 2018. Women were excluded from the analysis at time points where they were not at risk of an unintended pregnancy. Latent status membership probabilities, item-response probabilities, transitions probabilities and the effect of predictors on latent status membership were estimated and reported.

**Results:**

Patterns of contraceptive use were relatively consistent over time, particularly for high efficacy contraceptive methods with 71% of women using long-acting reversible contraceptives in 2012 also using long-acting reversible contraceptives in 2018. Multiple contraceptive use was highest in 2006 when women were aged 28–33 years (19.3%) but declined over time to 14.3% in 2018 when women were aged 40–45 years. Overall, contraceptive patterns stabilised as the women moved into their late 30s and early 40s.

**Conclusions:**

Although fertility declines with age, the stability of contraceptive choice and continued use of short-acting contraception among some women suggests that a contraceptive review may be helpful for women during perimenopause so that they are provided with contraceptive options most appropriate to their specific circumstances.

## Introduction

In Australia, the rate of unintended pregnancy has been estimated to be as high as 40% with around one-third of these pregnancies ending with termination [1]. Previous research from the U.S. has indicated that rates of unintended pregnancy are highest among women aged under 25 years, followed by women aged 40–44 years [2]. Research in more recent years has suggested that the rate of unintended pregnancy is increasing for older women [3]. An Australian population-based survey found that 43% of respondents who reported an unintended pregnancy in the previous ten years were aged over 30 years [1]. Although women are deferring pregnancy to later ages [4], these studies highlight a potential lack of adequate contraception counselling for women heading into their later reproductive years (including those in perimenopause).

To date, much of the research aimed at understanding contraceptive patterns has focused on younger women [5–8] or relied on cross-sectional data that takes a snapshot of contraceptive use at one point in time [9–12]. However, previous work in the U.S. found that women aged 35–44 years were 3.2 times more likely to be non-users of contraception compared to women aged 18–24 years over a 12 month period [9]. This suggests that contraceptive use is not stable across age. In addition, among older women who were contraceptive users, 12% reported having a gap in contraception despite being at risk of an unintended pregnancy. However, only a small number of studies have examined longitudinal patterns of contraception using nationally representative data [6, 13–16], with few including women aged over 30 [14, 16]. No studies have focused on contraceptive combinations among women of older reproductive age, with samples often focused on either single methods or a primary method (such as the pill) combined with barrier methods for STI protection [14, 17]. In an Australian cross-sectional study involving women aged 40–44 years in 2005, 26% reported using the contraceptive pill only, 16% reported condom only, 7% reported using long-acting reversible contraceptive (LARC) methods only and 3% reported multiple contraception methods [17]. Studies from other high income countries suggest that multiple method use may be much higher [9, 18, 19] while a recent Australian study focused on women aged 18–23 years showed the use of 30 distinct contraceptive patterns at the time of last vaginal sex [7].

How women actually use contraception as they transition through and out of their main childbearing years and what factors influence these transitions is unknown. Further, women’s risk of unintended pregnancy has been shown to be dynamic, and in order to accurately reflect contraceptive behaviour, reasons for non-contraceptive use also need to be considered [16]. In previous contraceptive research, sociodemographic and reproductive health factors have largely been explored as contributing factors [7, 11, 12, 17, 20].

To better understand the contraceptive practices of women as they transition through their childbearing years and move towards menopause, this study examined patterns of contraceptive use and contraceptive transitions over time among an Australian cohort of women born 1973–78 who have been prospectively followed for over 20 years. This study also examined baseline characteristics (when aged 28 to 33 years) that may influence contraceptive choices.

## Materials and methods

### Study design

The Australian Longitudinal Study on Women’s Health (ALWSH) is a prospective cohort study focussed on the health of Australian women. Surveys began in 1996 and collects data on women’s demographics, physical and mental health, lifestyle and use of health services. Further information on the study has been detailed elsewhere [21] and is available at www.alswh.org.au. All data for this project were obtained from the ALSWH and approved under their Expression of Interest process (EOI A787) and provided in de-identified form. Ethics approval was granted from the University of Newcastle and the University of Queensland’s Human Research Ethics Committees. All participants provided informed written consent.

### Participants

**Fig 1:**
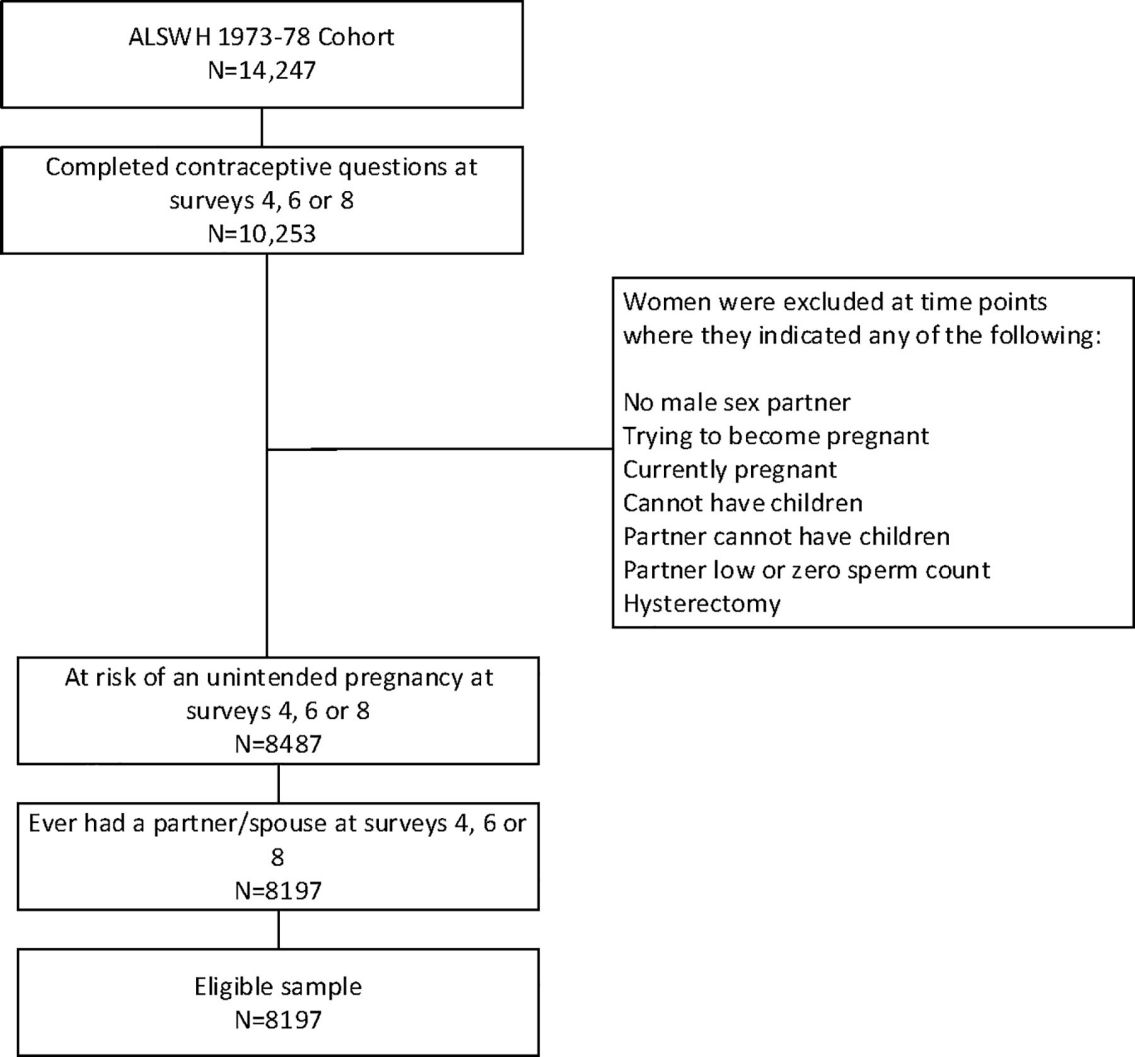
Determination of eligible sample.

This analysis focused on women born between 1973 and 1978 (known as the 1973–78 cohort). The 1973–78 cohort was first surveyed in 1996 when aged 18–23 years (N = 14,247), then in 2000 (aged 22–27 years), and every three years thereafter until 2018. Given that the focus is on contraceptive transitions as women move through their main reproductive years and towards menopause, women were eligible for this study if: (i) they completed the detailed contraceptive questions included at Survey 4 (2006, aged 28–33 years), Survey 6 (2012, aged 34–39 years) or Survey 8 (2018, aged 40–45 years); (ii) were at risk of an unintended pregnancy at any of the same surveys; and (iii) indicated they had ever had a partner at any of the same surveys (in order to be able to assess the relationship between violence and contraceptive use). Women were considered not at risk of an unintended pregnancy if they reported that they did not have a male sexual partner, were pregnant at the time of survey completion, were trying to become pregnant, had undergone a hysterectomy, they or their partner were not able to have children, or if their partner had low or zero sperm count. Based on the eligibility criteria, 8,197 women were selected for analysis in this study (Fig 1). It is important to note that the risk of unintended pregnancy was dynamic with 2,365, 2,308 and 1,805 women at Surveys 4, 6 and 8 respectively deemed to be not at risk of an unintended pregnancy.

Surveys 4, 6 and 8 were selected for analysis because (i) these time points cover the main reproductive years for many Australian women, (ii) detailed contraceptive questions were included in the ALSWH surveys from Survey 4 onwards, and (iii) three time points allowed for meaningful modelling of transitions without overcomplicating the interpretation. Only eight women at Survey 4 (0.09%), 47 women at Survey 6 (0.59%), and 261 women at Survey 8 (3.67%) did not complete the contraceptive questions.

### Measures

#### Contraceptive use

Women responded to the question “What forms of contraception do you use now?”, where they were able to indicate one or more of the following 14 responses options: “a combined oral contraceptive pill (The Pill)”, “a progestogen-only oral contraceptive pill (The Mini Pill)”, “an oral contraceptive pill but I do not know what type”, “condoms”, “emergency contraception (e.g. morning after pill)”, “an implant (e.g. Implanon)”, “the withdrawal method”, “a copper intrauterine device”, “a progestogen intrauterine device (e.g. Mirena)”, “an injection (e.g. Depo-Provera)”, “a safe period method (e.g. natural family planning, rhythm method, Billings method, body temperature method, periodic abstinence)”, “a vaginal ring (e.g. Nuvaring)”, “another method of contraception” and “no contraception”. In separate questions, women were asked to indicate whether their partner had had a vasectomy or whether they had had a tubal sterilisation.

Based on previous research, response options were initially collapsed into eight groups based on contraceptive efficacy [7]: sterilisation (vasectomy or tubal sterilisation); LARCs, (the progestogen intrauterine system, the copper intrauterine device and the implant); short-acting hormonal contraceptives (progestogen-only contraceptive pill, combined oral contraceptive pill, oral contraception of unknown type, vaginal ring and the injectable); condoms; natural methods (withdrawal and fertility awareness methods); emergency contraception; other (another method of contraception); and no contraception.

### Covariates

The final models included covariates which were hypothesised *a priori* to be associated with contraceptive use.

Intimate partner violence (IPV) was measured using the Composite Abuse Scale (CAS) [22], which has been previously adapted and validated in this cohort of Australian women [23]. The modified form of the Community CAS (CCAS) used in the 1973–78 cohort is composed of 28 items that address physical, emotional, harassment and sexual abuse domains. History of IPV was coded as a dichotomous variable, with women who responded yes to any of the 28 CCAS items considered to have ever experienced IPV from that time point thereafter.

Demographic variables included age (in 2006), highest qualification (no formal qualification, school certificate/higher school certificate, trade/apprenticeship/certificate/diploma, and university degree/higher university degree), area of residence classified according to ARIA+ score (major cities, inner/outer regional, and remote/very remote), relationship status (partnered and unpartnered) and employment status (full-time, part-time, or not in paid work). Health care card status was included as a surrogate measure of socioeconomic status.

Health-related variables included smoking status (current smoker, ex-smoker, and non-smoker), alcohol consumption (low risk drinker, non-drinker, rarely drinks, and risky/high risk drinker) [24], history of illicit drug use (ever/never), and body mass index (BMI) based on World Health Organization guidelines (underweight [<18.5 kg/m^2^], healthy [≥18.5 and <25 kg/m^2^], overweight [≥25 and <30 kg/m^2^] and obese [≥30 kg/m^2^]). Due to low frequency of the underweight category, the underweight and healthy categories were combined for analysis purposes. Mental health was measured using the SF-36 mental health subscale score [25] with scores less than or equal to 72 treated as indicative of poor mental health while scores higher than 72 were used as the reference category [26].

Reproductive health variables included pregnancy history (yes or no) and history of pregnancy termination for any reason (yes or no). Parity was also measured and categorised as zero, one, two, and three or more.

### Statistical analyses

Descriptive statistics were used to describe observed contraceptive use at three time points (2006, 2012 and 2018) as well as sample characteristics at baseline (in 2006). Latent transition analysis (LTA, [27]) was employed across time to investigate combinations of contraceptives, changes in combinations of contraceptives over time, and baseline characteristics associated with combinations of contraceptives. Our exploration of LTA models evaluated between three to ten potential latent statuses in order to determine the optimal model. Each model had three time points: Survey 4 (Time 1, 2006), Survey 6 (Time 2, 2012) and Survey 8 (Time 3, 2018). The optimal model was selected based on the likelihood ratio statistic (G^2^), the Akaike Information Criterion (AIC) [28], the Bayesian Information Criterion (BIC), along with meaningful clinical interpretability. We also sought to avoid including latent statuses with membership probabilities of less than 2% as this could lead to numerical problems when estimating the odds of latent status membership conditional on covariates. Once the optimal model was selected, we describe the latent statuses (which are unobserved) to reflect the combinations of contraceptives that best fit the data, as well as describing the probability of transitioning between latent statuses over time.

The optimal LTA model was extended to include covariates to estimate the odds of latent status membership at Time 1. Each covariate was entered into a separate LTA model to estimate the association between the covariate and contraceptive use. Estimating the effect of covariates in an LTA framework was restricted to Time 1 when women were aged 28–33 years. This approach is preferable to using three time points with a classify-analyse approach, which does not account for the uncertainty in status classification and thereby reduces the reliability of any inferences [27].

The LTA software used for this analysis did not support calculation of standard errors and confidence intervals at the time of publication. Consequently, we employed similar methods to O’Neill et al. [29] in using a bootstrap approach to obtain confidence intervals for the odds ratio estimates. We obtained 500 bootstrap datasets by sampling with replacement, and the LTA model with covariates was applied to each dataset. This allowed standard errors and confidence intervals to be estimated from the resulting bootstrap distribution.

We report the final model’s estimates of the latent status membership probabilities, the item-response probabilities, the transition probabilities, and the odds of latent status membership condition on covariates. All models used the PROC LTA procedure (The Methodology Centre, Penn State) available as a plugin to SAS 9.4 software (SAS Institute Inc., Cary, NC).

## Results

### Sample characteristics

When ALSWH first recruited in 1996, the women aged 18–23 years at the time were broadly representative of Australian women of the same age, with slight over-representation of women with tertiary qualifications. The analysed sample was broadly representative of the entire ALSWH 1973–78 cohort in 2006, with slight differences observed for history of IPV and relationship status (S1 Table). In 2006, when the women in the sample were aged 28–33 years, around one in four women had reported a history of IPV and one in six women were currently without a partner. Two-thirds of the women were drinking within the recommended guidelines, one in five women were current smokers and four in ten women did not participate in the recommended amount of physical activity. Eight in ten women were in paid work, with three in ten women working part-time.

### Contraceptive use

Short-acting contraception and condoms were the most common forms of contraception across all three time points, with initial proportions in 2006 of 45.7% and 31.3%, respectively, but these proportions decreased over time (Table 1). Low use of LARCs was reported in 2006 (5.6%) when women were aged 28–33 years, but this increased to 21.5% by 2018 when they were aged 40–45 years. A similar trajectory was noted for the use of sterilisation methods. Use of natural methods was relatively stable over time, only decreasing from 12.9% in 2006 to 11.2% in 2018. Use of other methods was not common at any time point, decreasing from 9.1% in 2006 to 4.0% in 2018. The proportion of women who reported using no contraception increased slightly over time (6.5% in 2006 to 11.9% in 2018). Emergency contraception was reported with very low frequencies across the three time points (0.3% to 1.2%). Given these low frequencies and the role of emergency contraception being to prevent pregnancy where contraception has not been used, misused or has failed, emergency contraception was excluded from the response items used in the LTA analyses.

**Table 1:** Observed contraceptive use at each survey.

Contraceptive groups		Survey 4 (2006) Aged 28–33	Survey 6 (2012) Aged 34–39	Survey 8 (2018) Aged 40–45
		N = 5387	N = 5211	N = 4686
		n (%)	n (%)	n (%)
Condom		1688 (31.3%)	1364 (26.2%)	819 (17.5%)
Short-acting contraception^A^		2463 (45.7%)	1484 (28.5%)	718 (15.3%)
LARCs^B^		300 (5.6%)	745 (14.3%)	1008 (21.5%)
Natural methods^C^		695 (12.9%)	695 (13.3%)	527 (11.2%)
Sterilisation methods^D^		527 (9.8%)	1160 (22.3%)	1542 (32.9%)
Other methods		489 (9.1%)	165 (3.2%)	189 (4.0%)
Emergency contraception		63 (1.2%)	36 (0.7%)	15 (0.3%)
No contraception		350 (6.5%)	495 (9.5%)	581 (12.4%)
Number of contraceptives	0	350 (6.5%)	495 (9.5%)	581 (12.4%)
	1	3995 (74.2%)	3882 (74.5%)	3435 (73.3%)
	2	962 (17.9%)	778 (14.9%)	646 (13.8%)
	3	77 (1.4%)	51 (1.0%)	21 (0.4%)
	4	3 (0.1%)	4 (0.1%)	2 (<1%)
	5	0 (0.0%)	0 (0.0%)	1 (<1%)
	6	0 (0.0%)	1 (<1%)	0 (0.0%)

^A^ Across all three time points, the short-acting category was composed of the oral contraceptive pill (91.5%), minipill (5.8%), vaginal ring (0.8%), and injection (2.4%).

^B^ The long-acting reversible contraceptives (LARCs) category was composed of progestogen-only IUD (64.6%), implant (32.0%), and copper IUD (4.1%) across all time points.

^C^ The natural methods category was dominated by the withdrawal method (88.8%) and fertility awareness methods (20.2%).

^D^ The sterilisation category was dominated by vasectomy (80.0%) and tubal sterilisation (20.7%).

Note: Individual contraceptives that make up each category do not add to 100% due to respondents who reported more than one item.

### Contraceptive transitions

We selected the model with six latent statuses as the optimal model as this model was characterised by strong class separation, clear clinical interpretability, and reasonable values of G^2^, AIC and BIC (S2 Table). This model was used as the basis for all subsequent analyses. Each status was dominated by a single contraceptive method with very high probability, but in some cases additional contraceptive methods were also included with lower probabilities (Table 2). Note that the values presented in Table 2 relate to the latent (unobserved) contraceptive statuses, whereas the values presented in Table 1 are the observed contraceptive prevalences. The first status (denoted as “Natural with other”) was dominated by natural methods (78% probability) but also included other contraceptive methods (21% probability) with some condom use (12%). The second status (denoted as “Short-acting with condom”) was dominated by short-acting methods (100% probability) with slight use of condoms (11% probability). The third status (known as “Condom with natural”) was characterised by high condom use (100% probability) with some use of natural methods (16% probability). The fourth status was dominated by sterilisation methods (100% probability) with limited use of other contraceptive methods (16% probability). The fifth status was dominated by use of LARCs only, and the sixth status captured the absence of any active contraceptive methods.

**Table 2:** Item-response probabilities for the selected optimal six-status model.

		Item-response probabilities for each status						
Latent Status	Latent status description	Condom	Short-acting	LARCs	Natural	Other	Sterilisation	None
Status 1	Natural with other	0.12	0.04	0.00	0.78	0.21	0.01	0.00
Status 2	Short-acting with condom	0.11	1.00	0.00	0.03	0.00	0.01	0.00
Status 3	Condom with natural	1.00	0.08	0.01	0.16	0.00	0.02	0.00
Status 4	Sterilisation with other	0.02	0.04	0.06	0.01	0.16	1.00	0.00
Status 5	LARCs	0.03	0.01	1.00	0.00	0.00	0.01	0.00
Status 6	None	0.00	0.00	0.00	0.00	0.00	0.00	1.00

LARCs = Long-acting reversible contraceptives

When aged 28–33 years, nearly half of women (42%) were estimated to be using short-acting methods with some condom use, with nearly a quarter (24%) estimated to be using condoms with natural methods. A further 13% were estimated to be using the “natural with other” approach to contraception (Table 3). The probability of using LARCs was low when women were aged 28–33 years (5%), but this increased to 18% by the time women were aged 40–45 years in 2018. The estimated probability of using sterilisation with other forms of contraception was also low in 2006 (9%) but this probability increased substantially to 33% when women were aged 40–45 years in 2018. Correspondingly, the estimated probabilities decreased over time for women using short-acting methods with some condom use, or using condoms with natural methods, or using a predominantly natural approach to contraception.

**Table 3:** Latent status membership probabilities (delta estimates) for six-status model.

Latent Status	Latent status description	Time 1 (2006)	Time 2 (2012)	Time 3 (2018)
Status 1	Natural with other	0.13	0.10	0.09
Status 2	Short-acting with condom	0.42	0.25	0.13
Status 3	Condom with natural	0.24	0.21	0.15
Status 4	Sterilisation with other	0.09	0.21	0.33
Status 5	LARCs	0.05	0.13	0.18
Status 6	None	0.07	0.10	0.13

For the most part, women were most likely to stay in the same contraceptive status from one time point to the next. However, women tended to transition to higher efficacy contraception over time. Nearly half the women using LARCs in 2006 (48%) were predicted to use LARCs again in 2012, while nearly three-quarters of the women (71%) using LARCs in 2012 were predicted to be using the same contraception in 2018 (Table 4). Women using no contraception in 2006 had a very high probability (72%) that they would move to a different contraceptive strategy by 2012 (notably towards a predominantly natural approach with a 17% probability, or towards sterilisation with a 19% probability). However, if using no active contraceptive strategy in 2012, women were likely to still be doing that in 2018 (40% probability), although some women would still transition to natural methods (12%) or to sterilisation with other contraceptives (15%).

**Table 4:** Status transition probabilities from Time 1 (2006) to Time 2 (2012), and from Time 2 (2012) to Time 3 (2018).

**Time 1 (2006) to Time 2 (2012)**							
		Time 2 (2012, aged 34–39)					
		Natural with other	Short-acting with condom	Condom with natural	Sterilisation with other	LARCs	None
Time 1 (2006, aged 28–33)	Natural with other	0.32†	0.10	0.11	0.16	0.21	0.11
	Short-acting with condom	0.06	0.45†	0.13	0.14	0.11	0.11
	Condom with natural	0.06	0.12	0.53†	0.16	0.07	0.06
	Sterilisation with other	0.00	0.05	0.02	0.87†	0.03	0.04
	LARCs	0.04	0.15	0.11	0.17	0.48†	0.05
	None	0.17	0.11	0.15	0.19	0.12	0.28
**Time 2 (2012) to Time 3 (2018)**							
		Time 3 (2018, aged 40–45)					
		Natural with other	Short-acting with condom	Condom with natural	Sterilisation with other	LARCs	None
Time 2 (2012, aged 34–39)	Natural with other	0.50†	0.04	0.02	0.17	0.10	0.18
	Short-acting with condom	0.04	0.41†	0.09	0.17	0.14	0.14
	Condom with natural	0.05	0.05	0.51†	0.20	0.12	0.07
	Sterilisation with other	0.02	0.01	0.00	0.89†	0.02	0.05
	LARCs	0.03	0.04	0.02	0.15	0.71†	0.04
	None	0.12	0.10	0.11	0.15	0.12	0.40†

† transition probability ≥ 0.30

LARCs = Long-acting reversible contraceptives

It is important to note that women are only included in the LTA model at time points where they were at risk of an unintended pregnancy. Women are only assigned to a contraceptive latent status at a particular time point if they are at risk of an unintended pregnancy at that time point. Consequently, the transition probabilities presented in Table 4 are only calculated for women who were assigned a latent status at both relevant time points. For example, women with a particular latent status at Time 1 but who were not at risk at Time 2 will not have been included in the calculation of transition probabilities between Time 1 and Time 2.

### Predictors of contraceptive use when aged 28–33 years

When investigating which factors were associated with contraceptive status when aged 28–33 years, the status of using short-acting contraceptives with some condom use was chosen as the reference level as this was the most common status at baseline (latent status membership probability of 42%, where all women in this status were predicted to use short-acting contraceptives with an 11% probability of also using condoms).

Women who had a history of IPV had around 50% higher odds for using sterilisation with other contraceptive methods (OR = 1.57, 95% CI = 1.19, 2.03) or for using LARCs (OR = 1.52, 95% CI = 1.15, 2.00) and a 17% increase in odds for using a predominantly natural approach (OR = 1.17, 95% CI = 1.01, 1.38) when compared to women without a history of IPV and relative to use of short-acting contraceptives and condoms (Table 5).

**Table 5:** Odds ratio estimates of effect of baseline characteristics on latent contraceptive status membership at Time 1 (2006), with short-acting contraceptive methods with condoms selected as the default/reference contraceptive status.

		Natural with other	Condom with natural	Sterilisation with other	LARCs	None
Baseline characteristic (in 2006)	N (%)*	OR (95% CI)	OR (95% CI)	OR (95% CI)	OR (95% CI)	OR (95% CI)
**ABUSE**						
Intimate partner violence (IPV)						
No	3917 (72.7%)	Ref.	Ref.	Ref.	Ref.	Ref.
Yes	1291 (24.0%)	1.17 (1.01, 1.38)	1.19 (0.91, 1.48)	1.57 (1.19, 2.03)	1.52 (1.15, 2.00)	1.12 (0.88, 1.45)
**DEMOGRAPHICS**						
Age at baseline (years)		1.10 (1.06, 1.15)	1.29 (1.20, 1.38)	1.44 (1.31, 1.55)	1.13 (1.04, 1.22)	1.19 (1.11, 1.28)
Highest qualification						
No formal qualifications	1382 (25.7%)	Ref.	Ref.	Ref.	Ref.	Ref.
School/higher school cert.	1460 (27.1%)	1.08 (0.89, 1.29)	0.98 (0.74, 1.29)	0.62 (0.46, 0.84)	0.97 (0.71, 1.33)	0.92 (0.67, 1.25)
Trade/apprentice/cert./diploma	1729 (32.1%)	1.18 (0.99, 1.40)	0.53 (0.39, 0.70)	0.18 (0.11, 0.25)	0.62 (0.44, 0.87)	0.61 (0.44, 0.87)
University/higher degree	804 (14.9%)	1.18 (0.97, 1.46)	0.70 (0.47, 1.02)	0.13 (0.05, 0.20)	0.56 (0.34, 0.83)	0.76 (0.52, 1.07)
Area of residence						
Major cities	2976 (55.2%)	Ref.	Ref.	Ref.	Ref.	Ref.
Regional	2150 (39.9%)	0.78 (0.67, 0.90)	1.80 (1.42, 2.24)	3.01 (2.32, 3.94)	1.51 (1.17, 1.95)	0.90 (0.71, 1.14)
Remote	184 (3.4%)	0.71 (0.46, 1.12)	1.93 (1.07, 3.17)	3.90 (1.84, 6.19)	1.21 (0.44, 2.17)	1.17 (0.54, 1.98)
Current relationship status						
Partnered	4500 (83.5%)	Ref.	Ref.	Ref.	Ref.	Ref.
Unpartnered	874 (16.2%)	0.80 (0.67, 0.95)	0.48 (0.33, 0.64)	0.23 (0.13, 0.39)	0.84 (0.57, 1.18)	0.57 (0.43, 0.77)
Work status						
Not in paid work	1175 (21.8%)	Ref.	Ref.	Ref.	Ref.	Ref.
Part-time	1644 (30.5%)	0.65 (0.54, 0.78)	0.82 (0.63, 1.08)	0.81 (0.59, 1.13)	0.80 (0.55, 1.16)	0.51 (0.38, 0.71)
Full-time	2564 (47.6%)	0.49 (0.40, 0.58)	0.29 (0.22, 0.39)	0.20 (0.14, 0.27)	0.43 (0.30, 0.59)	0.26 (0.20, 0.34)
**HEALTH BEHAVIOURS**						
Smoking status						
Non-smoker	3099 (57.5%)	Ref.	Ref.	Ref.	Ref.	Ref.
Ex-smoker	1179 (21.9%)	1.19 (1.00, 1.40)	1.15 (0.87, 1.54)	1.47 (1.06, 2.00)	1.11 (0.76, 1.46)	1.33 (1.01, 1.84)
Current smoker	1087 (20.2%)	0.94 (0.80, 1.12)	1.44 (1.09, 1.88)	1.47 (1.05, 2.01)	1.24 (0.87, 1.68)	1.36 (1.00, 1.82)
Alcohol consumption						
Low risk drinker	3357 (62.3%)	Ref.	Ref.	Ref.	Ref.	Ref.
Non-drinker	453 (8.4%)	1.55 (1.21, 1.97)	2.19 (1.53, 3.10)	1.51 (0.90, 2.42)	1.77 (1.09, 2.57)	2.63 (1.77, 3.81)
Rarely drinks	1345 (25.0%)	1.09 (0.93, 1.29)	1.42 (1.08, 1.79)	1.57 (1.15, 2.06)	1.04 (0.72, 1.38)	1.75 (1.33, 2.22)
Risky/high risk drinker	203 (3.8%)	0.80 (0.57, 1.16)	1.29 (0.65, 2.12)	1.14 (0.52, 1.97)	1.39 (0.67, 2.35)	1.29 (0.62, 2.13)
Body mass index						
Healthy/underweight	3169 (58.8%)	Ref.	Ref.	Ref.	Ref.	Ref.
Overweight	1273 (23.6%)	1.02 (0.87, 1.19)	0.97 (0.71, 1.25)	1.41 (1.02, 1.84)	1.61 (1.21, 2.13)	1.27 (0.93, 1.65)
Obese	822 (15.3%)	0.99 (0.81, 1.17)	1.59 (1.14, 2.03)	2.41 (1.69, 3.27)	1.61 (1.13, 2.24)	1.56 (1.12, 2.06)
Ever used illicit drugs						
No	2045 (38.0%)	Ref.	Ref.	Ref.	Ref.	Ref.
Yes	3297 (61.2%)	1.10 (0.96, 1.26)	1.02 (0.82, 1.28)	0.73 (0.55, 0.94)	0.96 (0.76, 1.24)	1.29 (1.03, 1.68)
Poor mental health						
No	2999 (55.7%)	Ref.	Ref.	Ref.	Ref.	Ref.
Yes	2383 (44.2%)	1.08 (0.95, 1.24)	1.10 (0.87, 1.35)	1.18 (0.91, 1.48)	1.19 (0.93, 1.59)	0.90 (0.71, 1.13)
**REPRODUCTIVE FACTORS**						
Ever been pregnant						
No	2993 (55.6%)	Ref.	Ref.	Ref.	Ref.	Ref.
Yes	2388 (44.3%)	1.60 (1.40, 1.83)	0.71 (0.55, 0.93)	0.18 (0.12, 0.26)	0.85 (0.65, 1.09)	2.67 (2.05, 3.44)
Ever had a pregnancy termination						
No	4510 (83.7%)	Ref.	Ref.	Ref.	Ref.	Ref.
Yes	874 (16.2%)	1.13 (0.94, 1.38)	1.58 (1.20, 2.15)	1.28 (0.90, 1.76)	1.14 (0.79, 1.57)	1.46 (1.09, 1.95)
**HEALTH SERVICE USE**						
Health care card						
No	4687 (87.0%)	Ref.	Ref.	Ref.	Ref.	Ref.
Yes	697 (12.9%)	1.21 (0.97, 1.48)	2.05 (1.43, 2.69)	2.21 (1.51, 3.02)	2.55 (1.79, 3.36)	1.36 (0.96, 1.90)

* Note that column percentages may not add to 100% due to missing values (not shown); parity was not included due to collinearity with pregnancy history. LARC = Long-acting reversible contraceptives.

Increasing age was associated with increased odds of all contraceptive statuses when compared to the default status of short-acting methods used with condoms, with the strongest effect observed for sterilisation with other contraception choices (OR = 1.44, 95% CI = 1.31, 1.55).

Compared to women with no school qualifications, women with school qualifications had around 40% lower odds of using sterilisation with other contraceptives (OR = 0.62, 95% CI = 0.46, 0.84) while women with university qualifications had the lowest odds of using sterilisation with other contraceptives in 2006 when aged 28–33 years (OR = 0.13, 95% CI = 0.05, 0.20). We observed a similar association for sterilisation among women with trade/vocational qualifications (OR = 0.18, 95% CI = 0.11, 0.25). Women living in regional or remote areas had much higher odds of using sterilisation compared to their counterparts living in major cities (OR = 3.01 and 3.90, respectively).

Unpartnered women were less likely to be using a natural contraceptive approach, or to use condoms with natural methods compared to using short-acting contraceptive methods with condoms. Unpartnered women were also observed to have very low odds of sterilisation with other contraceptives (OR = 0.23, 95% CI = 0.13, 0.39) when compared with short-acting methods used with condoms. Working full-time was associated with lower odds of all statuses when compared to the default contraception combination of short-acting methods with condoms.

Current and ex-smokers generally had increased odds of using condoms with natural methods, sterilisation with other methods, and no contraception when compared to the default combination of short-acting contraceptive methods with condoms. Similarly, non-drinkers and rare drinkers generally had increased odds of all contraceptive statuses when compared to the default combination of short-acting methods with condoms, with particularly strong associations for non-drinking with use of condoms with natural methods (OR = 2.19, 95% CI = 1.53, 3.10) and no contraception (OR = 2.63, 95% CI = 1.77, 3.81).

A BMI in the obese category was associated with a more than two-fold increase in the odds of using sterilisation with other methods (OR = 2.41, 95% CI = 1.69, 3.27), an over 60% increase in the odds of using LARCs (OR = 1.61, 95% CI = 1.13, 2.24) as well as in the odds of no contraception (OR = 1.56, 95% CI = 1.12, 2.06). Women who had ever used illicit drugs were around 30% more likely to be using no contraception (OR = 1.29, 95% CI = 1.03, 1.68), while women who had never used illicit drugs were slightly less likely to use sterilisation (OR = 0.73, 95% CI = 0.55, 0.94).

Women who had ever been pregnant were more than twice as likely to not be using any contraception (OR = 2.67, 95% CI = 2.05, 3.44), had 60% increased odds of using natural contraceptive approaches with other methods (OR = 1.61, 95% CI = 1.40, 1.83), and had significantly lower odds of using sterilisation with other contraceptives (OR = 0.18, 95% CI = 0.12, 0.26). Women who had ever had a termination were more likely to be using condoms with natural methods (OR = 1.58, 95% CI = 1.20, 2.15) or no contraception (OR = 1.46, 95% CI = 1.09, 1.95), compared to the default of short-acting contraceptives with condoms. Women with a health care card were more than twice as likely to be using condoms with natural methods, LARCs or sterilisation, compared to women without a health care card.

## Discussion

### Main findings

Taking a life course approach to contraceptive use, this study extends current knowledge by examining contraceptive transitions as women move through their main childbearing years and towards menopause. By examining all contraceptive combinations for women who were at risk of an unintended pregnancy at each time point, we were able to provide a more accurate picture of contraceptive use and non-use among these women. Here, we found that contraceptive use was relatively stable across time, with short-acting hormonal contraception and condoms the most common contraceptive methods until the women reached 40–45 years when LARC and permanent contraception became most prevalent. Unlike young women who have been found to use multiple contraceptive methods at the same act of intercourse, we found that the majority of women employed one contraceptive method only, and when multiple methods were employed, they were likely to be of lower efficacy [7]. These findings have important implications for contraceptive counselling and reproductive life planning for women of older reproductive age.

### Interpretation

Overall, patterns of contraceptive use were found to be relatively consistent over time, particularly when contraceptive efficacy was high. In contrast to findings from Australian and international research that demonstrates frequent multiple method use among young women, our results demonstrate that as women move into their 30s and beyond, their contraceptive patterns appear to stabilise [7, 19]. Multiple contraceptive use in our study was highest in 2006 when aged 28–33 years at around 19%, with this decreasing over time to around 14% by age 40–45 years. This figure is similar to the cross-sectional examination of Wave 5 of the Household Income and Labour Dynamics in Australia (HILDA) survey [17]. We also found that when multiple methods were used, they were often of lower efficacy (e.g., condom combined with natural methods) although use of withdrawal as a contraceptive approach was lower than that found in other studies [19]. Withdrawal is often used as extra protection against pregnancy [19]; given the women in our study were transitioning through, and out of their main reproductive years and were typically in longer term relationships, they perhaps felt that any additional protection provided by withdrawal was unnecessary.

While LARC and permanent methods were found to be low in 2006 when the women were aged 28–33 years, they had the greatest increase in use over the observation period with around half of the cohort using these methods by age 40–45 years. Although our findings are consistent with the literature which has demonstrated a higher use of permanent contraceptive methods compared to LARC, in our study a higher proportion of women were using LARC methods when aged 40 and over [12]. The difference in findings may be due to the study timeframes with the ASHR2 data collected in 2012. Since this time, there has been increasing awareness in Australia regarding the acceptability of LARC across the reproductive life course and increasing its use among women is now recognised as a key indicator in meeting the priorities of the current National Women’s Health Strategy [30]. These strategies appear to have had some success. An analysis of the Pharmaceutical Benefits Scheme (PBS) data suggests that rates of hormonal LARC use doubled between 2006 to 2018, with the highest uptake among women aged 35–44 [31]. Given that women are deferring pregnancy to later ages and the interval between family completion and menopause is becoming shorter, LARC may be a more favoured option than sterilisation, with IUDs presenting few contraindications for perimenopausal women [32, 33].

Consistent with previous research, we found that women from regional and remote areas were earlier adopters of both LARC and sterilisation [20]. Those that reported either of these approaches were highly likely to continue to report using these methods across the observation period. Interestingly, 80% of sterilisations reported in our sample were via partner vasectomy. Although vasectomy is recommended due to being a safer procedure and less prone to failure as compared to female sterilisation, our findings contrast to other Australian research which found that partner vasectomy declined over time (although it increased within the sterilisation category) and was less likely to be used by those in remote areas [12, 34]. The high proportion of vasectomy is encouraging as women who were in the obese BMI category at baseline were also found to be more likely to use permanent methods of contraception. Women with a BMI over 30 have been found to be at increased risk of complications during laparoscopic sterilisation [34].

LARC use was also found to be higher among women who reported being in the overweight or obese BMI category when aged 28–33 years in 2006. While clinical trials on contraceptive efficacy often exclude these women, an analysis of the contraceptive CHOICE Project in the U.S. found that the three-year failure rates of IUDs (both progestogen and copper-based) in women who were overweight and obese were less than 1 per 100 women-years and failure rates for the contraceptive implant were even lower [35, 36]. Given the increasing rates of obesity and obesity-related chronic disease in Australia among women of reproductive age, guidance and support regarding safe and effective contraception for these women is paramount due to the high risk of pregnancy-related complications associated with obesity [37]. While there is inconsistent evidence regarding the effectiveness of short-term hormonal contraception in preventing unintended pregnancies among obese women, the development of additional cardiovascular risk profiles has been found to complicate their use and high-quality studies point to the combined oral contraceptive pill being less forgiving of inconsistent use among women with obesity, compared to those of a healthy weight [38–41].

Around one-quarter of women in the current study reported a history of IPV by age 28–33 years in 2006, with the experience of violence increasing significantly during their childbearing years [42]. Women who had a history of IPV at baseline had more than 50% higher odds of using LARC and sterilisation. Similar findings have been reported elsewhere. A U.S. study found that while women with a history of IPV were more likely to use the most effective contraceptive methods as compared to women with no history of IPV, patterns of contraceptive use varied by length of IPV exposure [43]. Women reporting recent IPV exposure were more likely to report emergency contraceptive use, and switching their usual method of contraception, while women with the longest exposure to IPV were more likely to report the use of ‘hidden’ contraceptive methods, like LARC and sterilisation. Prior Australian research has found that women who have experienced IPV were more likely to make autonomous contraceptive decisions, not use contraception at all or have their partner make the decisions than to make joint decisions [44]. Here, while women who had a history of IPV at baseline were most likely to use high efficacy methods, they also had a 17% increase in odds of using natural methods, demonstrating the complexity of contraceptive use in the context of IPV [45]. Women experiencing reproductive coercion clearly have specific contraceptive requirements, depending on the level of surveillance and monitoring they are experiencing [45]. Further research is therefore required to unpack the relationship between IPV and changes in contraceptive use across the reproductive life course.

Despite the use of short-acting hormonal contraception and condom use declining over time, by age 40–45 years 13% and 17% of women were still using these methods, respectively. These findings are comparable to those reported in the ASHR2 for women aged over 40 [12]. Given the often long and difficult process of finding ‘contraceptive fit’ that women report experiencing in their 20’s, these women potentially continue to return to methods they know work for them [46]. Additionally, while women who engaged in lower efficacy contraceptive methods when aged 28–33 years were likely to continue to use these approaches over time, these women also had a 20% predicted likelihood of transitioning to no contraception when aged 40–45 years despite being at risk of an unintended pregnancy. Likewise, although non-contraceptive users at Time 1 in 2006 had a high probability of transitioning to some form of contraception (either natural methods or sterilisation), these women also had a 43% predicted probability of remaining as non-contraceptive users at time point 3. At Survey 8 when the women were aged 40–45 years 12% of women who were at risk of a future unintended pregnancy were not using contraception. Non-contraceptive use in this cohort is more than double that reported in the ASHR2 and slightly higher than that reported internationally [12, 47]. A study conducted in the US, for example showed that women aged 35–44 years were 3.2 times more likely to be non-users of contraception for the entire 12-month observation period compared to women aged 18–24 years [9]. In addition, of the women of older reproductive age who were contraceptive users, 12% reported having a gap in contraception despite being at risk of an unintended pregnancy.

Taken together, these findings point to a significant issue regarding the need for the provision of contraceptive care for women of older reproductive age. Short-acting hormonal contraception such as the combined oral contraceptive pill (which drove short-acting contraception in this study) and condoms have been found to be the prime methods used at the time of an unintended pregnancy and this pattern has been found to persist for women across the reproductive life course [1, 5]. Both women and men of all ages hold misperceptions surrounding fertility and perhaps this is a driver of inconsistent effective contraceptive use when aged over 40, although further research is warranted [48]. While women are deferring pregnancy to later ages and the median age of menopause is 51 years, pregnancy is still possible for women over 40 despite lower fecundity and time to conception compared to younger women [49]. Pregnancies over the age of 40 carry significant maternal and neonatal risks including increased risk of chromosomal abnormalities, miscarriage and premature delivery as compared to younger women [50]. Although Australia lacks formal guidelines around the provision of contraception in later reproductive years, guidelines developed by the Faculty of Sexual and Reproductive Healthcare in the UK suggest that women over the age of 40 should be informed that effective contraception is still required until after menopause to prevent unintended pregnancies [51].

In addition, women of older reproductive age may experience perimenopausal symptoms that could be effectively managed with contraceptives [33]. We therefore support Bateson and McNamee’s recommendation regarding the importance of contraceptive review for safety and appropriateness during perimenopause [33]. Despite their high efficacy and suitability for most women (including perimenopausal women), there are currently limited LARC options available on the Australian market; only the 52 mg levonogestrel IUD and etonogestrel subdermal implant are subsidised under Australia’s PBS (a 19.5 mg levonogestrel IUD was introduced onto the PBS in March 2020, although its lower hormone dose and smaller size is considered a particular benefit for young, nulliparous users). A copper IUD is also available, however this method is not subsidised and women are required to bear the cost (approximately AUD 100), despite this method being not only the most effective non-hormonal method available, but also a method suitable for women of advanced reproductive age and those that have contraindications to hormonal contraception (e.g., breast cancer) [33, 51]. We add our support to calls for the copper IUD to be subsidised in Australia to facilitate greater uptake among those who would benefit from its use [52].

Alongside availability, choice of contraceptive method has been found to be linked to several factors, including lack of knowledge of LARC among both women and their providers [53–55]. Ensuring women have access to accurate information regarding all their contraceptive options is vital to facilitating contraceptive choice, as is providing education for general practitioners who are often the first point of contact for contraceptive advice [56]. Further compounding access issues, a lack of clinical training in LARC insertion has also been identified as a barrier to increasing LARC use [56]. Increasing LARC training among clinicians, including through the implementation of nurse (and midwife)-led models of care, is crucial to meeting the sexual and reproductive health goals of women across their reproductive life course. Research has demonstrated the suitability of registered nurses and midwives as well as nurse/midwife practitioners to undertake contraception and family planning services including LARC insertion and removal [56]. With LARC as an indicator for the success of priority 1 of the 2020–2030 Women’s Health Strategy, funding models that include Medicare provider numbers for nurses and midwives may assist with increasing access and affordability of LARC for women of all ages [57].

### Strengths and limitations

The longitudinal nature of the data and ability to examine women’s contraceptive patterns over time is a particular strength of the study. In addition, we were able to examine a comprehensive set of contraceptive methods and apply statistical techniques to accurately identify contraceptive combinations. This improves on previous research which has often examined contraception using hierarchical approaches (highest efficacy) or applied pre-determined contraceptive patterns [5, 6, 9–11]. A further strength our study was that we accounted for women not being at risk of an unintended pregnancy at each of the time points. Here, we found 4,806 women (49.6%) were not at risk of an unintended pregnancy for at least one of the time points across the observation period and these women moved in and out of being ‘at risk’ depending on the time point. While researchers acknowledge the need to restrict samples to those at risk of unintended pregnancy, few have accounted for the dynamic nature of risk in longitudinal research [10, 16, 58].

Despite this, our study had a few limitations. Firstly, contraceptive use was examined at three time points, six years apart. We were therefore not able to identify contraceptive switching between these periods. Although evidence suggests younger women switch their contraceptive methods relatively frequently, few have explored the frequency of method switching among older reproductive aged women [59]. However, findings presented here suggest a stabilisation in contraceptive method use as women age. Additionally, a limitation of the LTA software was that we were unable to simultaneously adjust for multiple covariates. As a result, each covariate was entered into separate models. It is possible that this approach may have introduced some confounding between covariates, thereby biasing the estimates of baseline characteristics on latent status membership at time 1. Given that women had to have been at risk of an unintended pregnancy at one or more of the time points examined, only 58% of the original cohort (N = 14,247) who completed the baseline survey in 1996 were included in the analysis. However, these women were found to be largely comparable on sociodemographic and health factors to the original cohort (which was found to be demographically representative of similarly-aged women in the Australian population except for over-representation of tertiary educated and Australian-born women); and is one of the largest samples examining contraceptive transitions over time. As we were focused on women who were at risk of a future unintended pregnancy, we acknowledge that some women who were ineligible due to pregnancy may have experienced an unintended pregnancy. Likewise, we cannot discount that some women who indicated having no sexual partner may have engaged in casual sex and did not disclose this. It is also possible that some women may have undergone non-surgical early menopause and were therefore not at risk of an unintended pregnancy, but this information was not available in the survey data. Lastly, as we dichotomised the history of IPV at each time point into yes or no, we did not capture the full nuance of these experiences which may have influenced contraceptive decision-making.

## Conclusion

Extending on current knowledge regarding contraceptive use among women as they move through their reproductive years and towards menopause, this study showed that contraceptive use stabilises over time, with women often switching to more effective methods as women age. We also demonstrated that contraceptive use varies by sociodemographic characteristics, with geographical location, obesity and history of IPV all impacting contraceptive choices. Although fertility declines with age, the stability of contraceptive choice and continued use of short-acting contraception among some women suggests that a contraceptive review may be helpful for women during perimenopause so that they are provided with contraceptive options most appropriate to their specific circumstances.

## Supporting information

S1 Table Characteristics for sample of Australian women* aged 28–33 in 2006. (DOCX)

S2 Table Comparison of LTA models with 3–10 latent statuses with model fit statistics. (DOCX)

## References

[1] Taft AJ, Shankar M, Black KI, Mazza D, Hussainy S, Lucke JC. Unintended and unwanted pregnancy in Australia: a cross-sectional, national random telephone survey of prevalence and outcomes. Med J Aust. 2018;209(9):407–8. doi: 10.5694/mja17.01094 30282564

[2] Finer LB, Zolna MR. Unintended pregnancy in the United States: incidence and disparities, 2006. Contraception. 2011;84(5):478–85. doi: 10.1016/j.contraception.2011.07.013 22018121 PMC3338192

[3] Finer LB, Zolna MR. Shifts in intended and unintended pregnancies in the United States, 2001–2008. Am J Public Health. 2014;104 Suppl 1:S43–8. doi: 10.2105/AJPH.2013.301416 24354819 PMC4011100

[4] Australian Institute of Health and Welfare. Australia’s mothers and babies 2018: in brief. Canberra: AIHW, 2020.

[5] Coombe J, Harris ML, Wigginton B, Lucke J, Loxton D. Contraceptive use at the time of unintended pregnancy: findings from the Contraceptive Use, Pregnancy Intention and Decisions study. Aust Fam Physician. 2016;45(11):842–8. 27806456

[6] Rowlands IJ, Mishra GD, Lucke JC. Association between young women’s physical and mental health and their method of contraception in a longitudinal, population-based study. BMJ Sex Reprod Health. 2020. doi: 10.1136/bmjsrh-2019-200479 32522842

[7] Harris ML, Coombe J, Forder PM, Lucke JC, Bateson D, Loxton D. Young women’s complex patterns of contraceptive use: findings from an Australian cohort study. Perspect Sex Reprod Health. 2020;52(3):181–90. doi: 10.1363/psrh.12158 33191577

[8] Marshall C, Guendelman S, Mauldon J, Nuru-Jeter A. Young women’s contraceptive decision making: do preferences for contraceptive attributes align with method choice? Perspect Sex Reprod Health. 2016;48(3):119–27. doi: 10.1363/48e10116 27490460

[9] Frost JJ, Singh S, Finer LB. U.S. women’s one-year contraceptive use patterns, 2004. Perspect Sex Reprod Health. 2007;39(1):48–55. doi: 10.1363/3904807 17355381

[10] Frost JJ, Darroch JE. Factors associated with contraceptive choice and inconsistent method use, United States, 2004. Perspect Sex Reprod Health. 2008;40(2):94–104. doi: 10.1363/4009408 18577142

[11] Frost JJ, Singh S, Finer LB. Factors associated with contraceptive use and nonuse, United States, 2004. Perspect Sex Reprod Health. 2007;39(2):90–9. doi: 10.1363/3909007 17565622

[12] Richters J, Fitzadam S, Yeung A, Caruana T, Rissel C, Simpson JM, et al. Contraceptive practices among women: the second Australian study of health and relationships. Contraception. 2016;94(5):548–55. doi: 10.1016/j.contraception.2016.06.016 27373543

[13] Stidham Hall K, Moreau C, Trussell J, Barber J. Young women’s consistency of contraceptive use—does depression or stress matter? Contraception. 2013;88(5):641–9. doi: 10.1016/j.contraception.2013.06.003 23850075 PMC3796023

[14] Lucke JC, Watson M, Herbert D. Changing patterns of contraceptive use in Australian women. Contraception. 2009;80(6):533–9. doi: 10.1016/j.contraception.2009.05.122 19913147

[15] Hall KS, Moreau C, Trussell J, Barber J. Role of young women’s depression and stress symptoms in their weekly use and nonuse of contraceptive methods. J Adolesc Health. 2013;53(2):241–8. doi: 10.1016/j.jadohealth.2013.02.009 23582524 PMC3713141

[16] Jones RK, Tapales A, Lindberg LD, Frost J. Using longitudinal data to understand changes in consistent contraceptive use. Perspect Sex Reprod Health. 2015;47(3):131–9. doi: 10.1363/47e4615 26287965 PMC4976085

[17] Parr N, Siedlecky S. Use of ’dual protection’ and other combinations of contraceptive methods in Australia. Aust N Z J Public Health. 2007;31(6):567–70. doi: 10.1111/j.1753-6405.2007.00145.x 18081579

[18] Spinelli A, Talamanca IF, Lauria L. Patterns of contraceptive use in 5 European countries. European Study Group on Infertility and Subfecundity. Am J Public Health. 2000;90(9):1403–8. doi: 10.2105/ajph.90.9.1403 10983197 PMC1447615

[19] Jones RK, Lindberg LD, Higgins JA. Pull and pray or extra protection? Contraceptive strategies involving withdrawal among US adult women. Contraception. 2014;90(4):416–21. doi: 10.1016/j.contraception.2014.04.016 24909635 PMC4254803

[20] Lucke JC, Herbert DL. Higher uptake of long-acting reversible and permanent methods of contraception by Australian women living in rural and remote areas. Aust N Z J Public Health. 2014;38(2):112–6. doi: 10.1111/1753-6405.12208 24690048

[21] Dobson AJ, Hockey R, Brown WJ, Byles JE, Loxton DJ, McLaughlin D, et al. Cohort profile update: Australian Longitudinal Study on Women’s Health. Int J Epidemiol. 2015;44(5):1547,a-f. doi: 10.1093/ije/dyv110 26130741

[22] Hegarty K, Sheehan M, Schofield C. A multidimensional definition of partner abuse: development and preliminary validation of the Composite Abuse Scale. J Fam Violence. J Fam Violence. 1999;14:399–415.

[23] Loxton D, Powers J, Fitzgerald D, Forder P, Anderson AE, Taft A, et al. The community composite abuse scale: reliability and validity of a measure of intimate partner violence in a community survey from the ALSWH. J Womens Health Issues Care. 2013;2(4). doi: 10.4172/2325-9795.1000115

[24] Pols RG, Hawks DV. Is there a safe level of daily consumption of alcohol for men and women? Canberra: National Health and Medical Research Council, 1992.

[25] Ware JE Jr., Sherbourne CD. The MOS 36-item short-form health survey (SF-36). I. Conceptual framework and item selection. Med Care. 1992;30(6):473–83. 1593914

[26] Hoeymans N, Garssen AA, Westert GP, Verhaak PF. Measuring mental health of the Dutch population: a comparison of the GHQ-12 and the MHI-5. Health Qual Life Outcomes. 2004;2:23. doi: 10.1186/1477-7525-2-23 15132745 PMC428585

[27] Collins L, Lanza S. Latent class and latent transition analysis Hoboken, New Jersey: John Wiley & Sons; 2010.

[28] Akaike H. A new look at the statistical model identification. IEEE Trans Automat Contr. 1974;19:716–23.

[29] O’Neill A, O’Sullivan K, O’Keeffe M, Walsh C, Purtill H. The change of pain classes over time: a latent transition analysis. Eur J Pain. 2020;24(2):457–69. doi: 10.1002/ejp.1502 31680381

[30] Australian Government Department of Health. National women’s health strategy: 2020–2030. Canberra: Commonwealth of Autralia, 2020.

[31] Grzeskowiak LE, Calabretto H, Amos N, Mazza D, Ilomaki J. Changes in use of hormonal long-acting reversible contraceptive methods in Australia between 2006 and 2018: A population-based study. Aust N Z J Obstet Gynaecol. 2020. doi: 10.1111/ajo.13257 33095452

[32] Australian Bureau of Statistics. Births, Australia Canberra: ABS; 2019 [updated 9/12/20; cited 2020 17th December]. Available from: https://www.abs.gov.au/statistics/people/population/births-australia/2019.

[33] Bateson D, McNamee K. Perimenopausal contraception: a practice-based approach. Aust Fam Physician. 2017;46(6):372–7. 28609592

[34] Lincoln E, McKay R, Schunmann C. Male and female sterilisation. Obstet Gynaecol Reprod Med. 2020;30(7):219–24.

[35] Darney P, Patel A, Rosen K, Shapiro LS, Kaunitz AM. Safety and efficacy of a single-rod etonogestrel implant (Implanon): results from 11 international clinical trials. Fertil Steril. 2009;91(5):1646–53. doi: 10.1016/j.fertnstert.2008.02.140 18423453

[36] Xu H, Wade JA, Peipert JF, Zhao Q, Madden T, Secura GM. Contraceptive failure rates of etonogestrel subdermal implants in overweight and obese women. Obstet Gynecol. 2012;120(1):21–6. doi: 10.1097/AOG.0b013e318259565a 22678035 PMC4043143

[37] Linne Y. Effects of obesity on women’s reproduction and complications during pregnancy. Obes Rev. 2004;5(3):137–43. doi: 10.1111/j.1467-789X.2004.00147.x 15245382

[38] Lopez LM, Bernholc A, Chen M, Grey TW, Otterness C, Westhoff C, et al. Hormonal contraceptives for contraception in overweight or obese women. Cochrane Database Syst Rev. 2016;(8):CD008452. doi: 10.1002/14651858.CD008452.pub4 27537097 PMC9063995

[39] Dragoman MV, Simmons KB, Paulen ME, Curtis KM. Combined hormonal contraceptive (CHC) use among obese women and contraceptive effectiveness: a systematic review. Contraception. 2017;95(2):117–29. doi: 10.1016/j.contraception.2016.10.010 27823942 PMC11283816

[40] Westhoff CL, Torgal AH, Mayeda ER, Stanczyk FZ, Lerner JP, Benn EK, et al. Ovarian suppression in normal-weight and obese women during oral contraceptive use: a randomized controlled trial. Obstet Gynecol. 2010;116(2 Pt 1):275–83. doi: 10.1097/AOG.0b013e3181e79440 20664386

[41] Edelman AB, Cherala G, Munar MY, Dubois B, McInnis M, Stanczyk FZ, et al. Prolonged monitoring of ethinyl estradiol and levonorgestrel levels confirms an altered pharmacokinetic profile in obese oral contraceptives users. Contraception. 2013;87(2):220–6. doi: 10.1016/j.contraception.2012.10.008 23153898 PMC3925642

[42] Loxton D, Dolja-Gore X, Anderson AE, Townsend N. Intimate partner violence adversely impacts health over 16 years and across generations: a longitudinal cohort study. PLoS One. 2017;12(6):e0178138. doi: 10.1371/journal.pone.0178138 28582406 PMC5459340

[43] Fantasia HC, Sutherland MA, Fontenot HB, Lee-St John TJ. Chronicity of partner violence, contraceptive patterns and pregnancy risk. Contraception. 2012;86(5):530–5. doi: 10.1016/j.contraception.2012.03.005 22520646

[44] Bauleni EM, Hooker L, Vally HP, Taft A. Intimate-partner violence and reproductive decision-making by women attending Victorian maternal- and child-health services: a cross-sectional study. Aust J Prim Health. 2018;24(5):422–7. doi: 10.1071/PY17183 30107139

[45] Grace KT, Anderson JC. Reproductive coercion: a systematic review. Trauma Violence Abuse. 2018;19(4):371–90. doi: 10.1177/1524838016663935 27535921 PMC5577387

[46] Coombe J, Harris ML, Loxton D. Examining long-acting reversible contraception non-use among Australian women in their 20s: findings from a qualitative study. Cult Health Sex. 2019;21(7):822–36. doi: 10.1080/13691058.2018.1519119 30612512

[47] Kopp Kallner H, Thunell L, Brynhildsen J, Lindeberg M, Gemzell Danielsson K. Use of contraception and attitudes towards contraceptive use in Swedish women—a nationwide survey. PLoS One. 2015;10(5):e0125990. doi: 10.1371/journal.pone.0125990 25992901 PMC4439158

[48] Monester J, Fisher J, Kirkman M, Rowe H, Holton S. ’If I had known the fertility health facts sooner…’ Knowledge gaps as a barrier to effective fertility management: findings from the understanding fertility management in contemporary Australia survey. Eur J Contracept Reprod Health Care. 2019;24(4):274–9. doi: 10.1080/13625187.2019.1625326 31204870

[49] Aitken RJ. Age, the environment and our reproductive future: bonking baby boomers and the future of sex. Reproduction. 2014;147(2):S1–S11. doi: 10.1530/REP-13-0399 24194569

[50] Frederiksen LE, Ernst A, Brix N, Braskhoj Lauridsen LL, Roos L, Ramlau-Hansen CH, et al. Risk of adverse pregnancy outcomes at advanced maternal age. Obstet Gynecol. 2018;131(3):457–63. doi: 10.1097/AOG.0000000000002504 29420406

[51] Faculty of Sexual and Reproductive Healthcare Clinical Effectiveness Unit. Contraception for women aged over 40 years. UK: FSRH 2017 (Amended September 2019).

[52] Bateson D, Harvey C, Trinh L, Stewart M, Black KI. User characteristics, experiences and continuation rates of copper intrauterine device use in a cohort of Australian women. Aust N Z J Obstet Gynaecol. 2016;56(6):655–61. doi: 10.1111/ajo.12534 27704541

[53] Coombe J, Harris ML, Loxton D. What qualities of long-acting reversible contraception do women perceive as desirable or undesirable? A systematic review. Sex Health. 2016. doi: 10.1071/SH15189 27467568

[54] Black K, Lotke P, Buhling KJ, Zite NB, and on behalf of the Intrauterine contraception for Nulliparous women: Translating Research into Action (INTRA) group. A review of barriers and myths preventing the more widespread use of intrauterine contraception in nulliparous women. Eur J Contracept Reprod Health Care. 2012;17(5):340–50. doi: 10.3109/13625187.2012.700744 22834648 PMC4950459

[55] Goldhammer DL, Fraser C, Wigginton B, Harris ML, Bateson D, Loxton D, et al. What do young Australian women want (when talking to doctors about contraception)? BMC Fam Pract. 2017;18(1):35. doi: 10.1186/s12875-017-0616-2 28298197 PMC5353872

[56] Mazza D, Bateson D, Frearson M, Goldstone P, Kovacs G, Baber R. Current barriers and potential strategies to increase the use of long-acting reversible contraception (LARC) to reduce the rate of unintended pregnancies in Australia: an expert roundtable discussion. Aust N Z J Obstet Gynaecol. 2017;57(2):206–12. doi: 10.1111/ajo.12587 28294293

[57] Botfield JR, Wright SM, Fenwick SE, Cheng Y. Training nurses in contraceptive implant procedures: implications for practice in Australia. Collegian. 2021;28(1):114–20. 10.1016/j.colegn.2020.04.005.

[58] Wu J, Meldrum S, Dozier A, Stanwood N, Fiscella K. Contraceptive nonuse among US women at risk for unplanned pregnancy. Contraception. 2008;78(4):284–9. doi: 10.1016/j.contraception.2008.04.124 18847575

[59] Coombe J, Harris ML, Loxton D. Motivators of contraceptive method change and implications for long-acting reversible contraception (non-)use: a qualitative free-text analysis. Sex Reprod Healthc. 2019;19:71–7. doi: 10.1016/j.srhc.2018.12.004 30928138

