# Supplementary material for: Contraceptive use among women through their later reproductive years: Findings from an Australian prospective cohort study
Source: PLoS One. 2021 Aug 11;16(8):e0255913. doi: 10.1371/journal.pone.0255913 (PMC8357106; doi:10.1371/journal.pone.0255913)
Supplement: S1 Table — (DOCX) [file pone.0255913.s001.docx]

**S1 Table. Characteristics for sample of Australian women.**

|  |  | **Entire cohort**  **2006**  **Aged 28-33**  **n=9145** | **Analysed sample**  **2006**  **Aged 28-33**  **n=5387** | **Analysed sample**  **2012**  **Aged 34-39**  **n=5211** | **Analysed sample**  **2018**  **Aged 40-45**  **n=4686** |
| --- | --- | --- | --- | --- | --- |
| **Characteristic** | **Category** | **n (%)** | **n (%)** | **n (%)** | **n (%)** |
| Ever reported IPV | No | 5736 (62.7%) | 3917 (72.7%) | 2413 (46.3%) | 1748 (37.3%) |
|  | Yes | 3079 (33.7%) | 1291 (24.0%) | 2599 (49.9%) | 2716 (58.0%) |
|  | Missing | 330 (3.6%) | 179 (3.3%) | 199 (3.8%) | 222 (4.7%) |
|  |  |  |  |  |  |
| Country of birth | Australia | 8058 (88.1%) | 4776 (88.7%) | 4636 (89.0%) | 4166 (88.9%) |
|  | Other ESB | 449 (4.9%) | 257 (4.8%) | 250 (4.8%) | 238 (5.1%) |
|  | Non-ESB | 603 (6.6%) | 336 (6.2%) | 302 (5.8%) | 267 (5.7%) |
|  | Missing | 35 (0.4%) | 18 (0.3%) | 23 (0.4%) | 15 (0.3%) |
|  |  |  |  |  |  |
| Area of residence | Major cities | 5133 (56.1%) | 2976 (55.2%) | 2855 (54.8%) | 2599 (55.5%) |
|  | Regional | 3584 (39.2%) | 2150 (39.9%) | 2040 (39.1%) | 1831 (39.1%) |
|  | Remote | 303 (3.3%) | 184 (3.4%) | 149 (2.9%) | 95 (2.0%) |
|  | Missing | 125 (1.4%) | 77 (1.4%) | 167 (3.2%) | 161 (3.4%) |
|  |  |  |  |  |  |
| Work status | Not in paid work | 1721 (18.8%) | 1175 (21.8%) | 1042 (20.0%) | 435 (9.3%) |
|  | Part-time | 2726 (29.8%) | 1644 (30.5%) | 2320 (44.5%) | 1773 (37.8%) |
|  | Full-time | 4683 (51.2%) | 2564 (47.6%) | 1841 (35.3%) | 2475 (52.8%) |
|  | Missing | 15 (0.2%) | 4 (0.1%) | 8 (0.2%) | 3 (0.1%) |
|  |  |  |  |  |  |
| Relationship status | Partnered (married/de facto) | 6587 (72.0%) | 4500 (83.5%) | 4608 (88.4%) | 4229 (90.2%) |
|  | Unpartnered | 2519 (27.5%) | 874 (16.2%) | 593 (11.4%) | 450 (9.6%) |
|  | Missing | 39 (0.4%) | 13 (0.2%) | 10 (0.2%) | 7 (0.1%) |
|  |  |  |  |  |  |
| Education | No formal qualifications | 2301 (25.2%) | 1382 (25.7%) | 952 (18.3%) | 641 (13.7%) |
|  | School/higher school certificate | 2515 (27.5%) | 1460 (27.1%) | 1419 (27.2%) | 1269 (27.1%) |
|  | Trade/apprenticeship/cert./diploma | 2897 (31.7%) | 1729 (32.1%) | 1734 (33.3%) | 1518 (32.4%) |
|  | University/higher degree | 1398 (15.3%) | 804 (14.9%) | 1066 (20.5%) | 1245 (26.6%) |
|  | Missing | 34 (0.4%) | 12 (0.2%) | 40 (0.8%) | 13 (0.3%) |
|  |  |  |  |  |  |
| Smoking status | Non-smoker | 5288 (57.8%) | 3099 (57.5%) | 3248 (62.3%) | 2945 (62.8%) |
|  | Ex-smoker | 2018 (22.1%) | 1179 (21.9%) | 1399 (26.8%) | 1301 (27.8%) |
|  | Current smoker | 1792 (19.6%) | 1087 (20.2%) | 559 (10.7%) | 432 (9.2%) |
|  | Missing | 47 (0.5%) | 22 (0.4%) | 5 (0.1%) | 8 (0.2%) |
|  |  |  |  |  |  |
| Alcohol consumption | Low risk drinker | 5467 (59.8%) | 3357 (62.3%) | 3144 (60.3%) | 2856 (60.9%) |
|  | Non-drinker | 948 (10.4%) | 453 (8.4%) | 500 (9.6%) | 432 (9.2%) |
|  | Rarely drinks | 2343 (25.6%) | 1345 (25.0%) | 1323 (25.4%) | 1050 (22.4%) |
|  | Risky / high risk drinker | 342 (3.7%) | 203 (3.8%) | 231 (4.4%) | 343 (7.3%) |
|  | Missing | 45 (0.5%) | 29 (0.5%) | 13 (0.2%) | 5 (0.1%) |
|  |  |  |  |  |  |
| Physical activity | Nil/Sedentary | 944 (10.3%) | 524 (9.7%) | 664 (12.7%) | 528 (11.3%) |
|  | Low | 3134 (34.3%) | 1838 (34.1%) | 1752 (33.6%) | 1319 (28.1%) |
|  | Moderate | 2103 (23.0%) | 1258 (23.4%) | 1155 (22.2%) | 1003 (21.4%) |
|  | High | 2706 (29.6%) | 1636 (30.4%) | 1409 (27.0%) | 1492 (31.8%) |
|  | Missing | 258 (2.8%) | 131 (2.4%) | 231 (4.4%) | 344 (7.3%) |
|  |  |  |  |  |  |
| Ever used illicit drugs | No | 3543 (38.7%) | 2045 (38.0%) | 2059 (39.5%) | 2041 (43.6%) |
|  | Yes | 5502 (60.2%) | 3297 (61.2%) | 3146 (60.4%) | 2637 (56.3%) |
|  | Missing | 100 (1.1%) | 45 (0.8%) | 6 (0.1%) | 8 (0.2%) |
|  |  |  |  |  |  |
| Body mass index | Healthy/underweight | 5210 (57.0%) | 3169 (58.8%) | 2680 (51.4%) | 1981 (42.3%) |
|  | Overweight | 2141 (23.4%) | 1273 (23.6%) | 1395 (26.8%) | 1360 (29.0%) |
|  | Obese | 1574 (17.2%) | 822 (15.3%) | 1074 (20.6%) | 1253 (26.7%) |
|  | Missing | 220 (2.4%) | 123 (2.3%) | 62 (1.2%) | 92 (2.0%) |
|  |  |  |  |  |  |
| Poor mental health | No | 5022 (54.9%) | 2999 (55.7%) | 2991 (57.4%) | 2515 (53.7%) |
|  | Yes | 4111 (45.0%) | 2383 (44.2%) | 2217 (42.5%) | 2169 (46.3%) |
|  | Missing | 12 (0.1%) | 5 (0.1%) | 3 (0.1%) | 2 (<1%) |
|  |  |  |  |  |  |
| Ever been pregnant | No | 4959 (54.2%) | 2993 (55.6%) | 568 (10.9%) | 364 (7.8%) |
|  | Yes | 4078 (44.6%) | 2388 (44.3%) | 4643 (89.1%) | 4321 (92.2%) |
|  | Missing | 108 (1.2%) | 6 (0.1%) | 0 (0.0%) | 1 (<1%) |
|  |  |  |  |  |  |
| Ever terminated a pregnancy | No | 7658 (83.7%) | 4510 (83.7%) | 4264 (81.8%) | 3748 (80.0%) |
|  | Yes | 1460 (16.0%) | 874 (16.2%) | 942 (18.1%) | 929 (19.8%) |
|  | Missing | 27 (0.3%) | 3 (0.1%) | 5 (0.1%) | 9 (0.2%) |
|  |  |  |  |  |  |
| Parity | Zero | 4747 (51.9%) | 2364 (43.9%) | 903 (17.3%) | 617 (13.2%) |
|  | One | 1918 (21.0%) | 1050 (19.5%) | 726 (13.9%) | 588 (12.5%) |
|  | Two | 1735 (19.0%) | 1375 (25.5%) | 2277 (43.7%) | 2144 (45.8%) |
|  | Three or more | 745 (8.1%) | 598 (11.1%) | 1305 (25.0%) | 1337 (28.5%) |
|  |  |  |  |  |  |
| Health care card | No | 7779 (85.1%) | 4687 (87.0%) | 4638 (89.0%) | 4260 (90.9%) |
|  | Yes | 1360 (14.9%) | 697 (12.9%) | 519 (10.0%) | 420 (9.0%) |
|  | Missing | 6 (0.1%) | 3 (0.1%) | 54 (1.0%) | 6 (0.1%) |
