# Supplementary material for: Contraceptive use among women through their later reproductive years: Findings from an Australian prospective cohort study
Source: PLoS One. 2021 Aug 11;16(8):e0255913. doi: 10.1371/journal.pone.0255913 (PMC8357106; doi:10.1371/journal.pone.0255913)
Supplement: S2 Table — (DOCX) [file pone.0255913.s002.docx]

**S2 Table. Comparison of LTA models with 3-10 latent statuses with model fit statistics**

| **Number of latent statuses** | **Number of iterations** | **G_­­­_­­^2^** | **AIC** | **BIC** | **Latent status with <2% probability** |
| --- | --- | --- | --- | --- | --- |
| 3 | 262 | 17879.01 | 17949.01 | 18194.41 | No |
| 4 | 147 | 11789.31 | 11899.31 | 12284.95 | No |
| 5 | 141 | 7733.75 | 7891.75 | 8445.66 | No |
| 6 | 347 | 4605.87 | 4819.87 | 5570.11 | No |
| 7 | 724 | 7010.37 | 7288.37 | 8262.97 | Yes |
| 8 | 3438 | 4301.87 | 4651.87 | 5878.89 | Yes |
| 9 | 4989 | 2394.53 | 2824.53 | 4332.01 | Yes |
| 10 | 3703 | 2485.76 | 3003.76 | 4819.74 | Yes |

Initially, an LTA model with three latent statuses was evaluated, and then each subsequent model was evaluated with an additional latent status included until a model with ten latent statuses was fitted. The models with eight, nine and ten latent statuses had the most optimal values of G^2^, AIC and BIC. However, these models resulted in latent statuses with very low probabilities and multiple statuses with similar patterns of item-response probabilities.
